# FRMD6 inhibits human glioblastoma growth and progression by negatively regulating activity of receptor tyrosine kinases

**DOI:** 10.18632/oncotarget.12148

**Published:** 2016-09-20

**Authors:** Yin Xu, Kaiqiang Wang, Qin Yu

**Affiliations:** ^1^ Department of Oncological Sciences Icahn School of Medicine at Mount Sinai, New York, NY 10029, USA

**Keywords:** FRMD6, glioblastoma, receptor tyrosine kinase, hippo signaling pathway, c-Met

## Abstract

FRMD6 is an Ezrin/Radixin/Moesin (ERM) family protein and a human homologue of *Drosophila* expanded (*ex*). *Ex* functions in parallel of *Drosophila* merlin at upstream of the Hippo signaling pathway that controls proliferation, apoptosis, tissue regeneration, and tumorigenesis. Even though the core kinase cascade (MST1/2-Lats1/2-YAP/TAZ) of the Hippo pathway has been well established, its upstream regulators are not well understood. Merlin promotes activation of the Hippo pathway. However, the effect of FRMD6 on the Hippo pathway is controversial. Little is known about how FRMD6 functions and the potential role of FRMD in gliomagenesis and glioblastoma (GBM) progression. We demonstrate for the first time that FRMD6 is down-regulated in human GBM cells and tissues and that increased FRMD6 expression inhibits whereas FRMD6 knockdown promotes GBM cell proliferation/invasion *in vitro* and GBM growth/progression *in vivo*. Furthermore, we demonstrate that unlike increased expression of merlin, which enhances the stress induced activation of the Hippo pathway, increased FRMD6 expression displays little effect on the pathway. In contrast, we show that FRMD6 inhibits activation of a couple of receptor tyrosine kinases (RTKs) including c-Met and PDGFR and their downstream Erk and AKT kinases. Moreover, we show that expression of constitutively active c-Met, the TPR-Met fusion protein, largely reverses the anti-GBM effect of FRMD6 *in vivo*, suggesting that FRMD6 functions at least partially through inhibiting activity of RTKs especially c-Met. These results establish a novel function of FRMD6 in inhibiting human GBM growth and progression and uncover a novel mechanism by which FRMD6 exerts its anti-GBM activity.

## INTRODUCTION

FRMD6 is an Ezrin/Radixin/Moesin (ERM) family protein and a human homolog of *Drosophila* expanded (*ex*) [1, 2]. *E*x functions in parallel of *Drosophila* merlin (*mer*) at upstream of the Hippo signaling pathway and activates the pathway [3–5]. Loss of *ex* leads to formation of hyperplastic imaginal discs and overgrown wings whereas *ex* overexpression in *Drosophila* wing and eye leads to reduced numbers of cells in the tissues [6, 7]. The Hippo signaling pathway is known to control organ size, proliferation, apoptosis, tissue regeneration, and tumorigenesis. Much of the pathway is conserved from *Drosophila* to mammals [8–10]. The core Hippo signaling kinase cascade, MST1/2-Lats1/2-YAP/TAZ, is relatively well understood whereas the upstream regulators of the pathway in mammalian cells are much less studied. Merlin, a tumor suppressor, is known to promote the Hippo pathway activation whereas the effect of FRMD6 on the pathway remains controversial. Two different studies showed that FRMD6 functions either through or independently of the Hippo pathway [1, 11]. There are limited studies of FRMD6. FRMD6 was found to serve as a tumor suppressor of human breast cancer cells [1] and FRMD6 knockdown induces the epithelial–mesenchymal transition (EMT) in mammary epithelial cells [11].

The most malignant grade IV astrocytoma is glioblastoma multiforme (GBM) [12]. Currently, there is no effective treatment for GBM. The estimated median survival of GBM patients is less than 15 months and less than 5% of GBM patients survives longer than 5 years [12, 13]. Poor prognosis of GBM is largely due to lack of clear understanding of the molecular pathogenesis of the disease. We previously showed that merlin promotes the stress-induced activation of the Hippo signaling pathway in GBM cells, sensitizes the response of GBM cells to chemotherapeutic agents, and inhibits GBM growth *in vivo* [14]. However, the effect of FRMD6 on GBM growth and progression and the molecular mechanism underlying its effect are unknown. We therefore investigated the role of FRMD6 in GBM growth and progression and in regulating the Hippo signaling pathway. Our results demonstrate that FRMD6 is down-regulated in human GBM cells and tissues comparing to their normal counterparts and that increased expression of FRMD6 inhibits whereas FRMD6 knockdown promotes GBM cell proliferation/invasion *in vitro* and growth/progression *in vivo*. In addition, we demonstrate that unlike increased expression of merlin, which enhances the stress-induced activation of the Hippo pathway, increased expression of FRMD6 has little effect on the stress-induced activation of the core Hippo pathway components, MST1/2, LATS1 and YAP. In contrast, FRMD6 was found to inhibit activation of a couple of receptor tyrosine kinases (RTKs) including c-Met and PDGFR and the downstream Erk and AKT kinases. Moreover, we show for the first time that expression of a constitutively active c-Met, the TPR-Met fusion protein, largely reverses the anti-GBM effect of FRMD6 *in vivo*, suggesting that FRMD6 functions through inhibiting activity of RKTs especially c-Met. These results establish a novel function of FRMD6 in inhibiting GBM growth and progression and uncover a novel mechanism by which FRMD6 exerts its anti-GBM activity.

## RESULTS

### Human GBM cells and tissues express lower levels of FRMD6

Analyses of the TCGA-GBM data through the cBioPortal for Cancer Genomics generated little information on FRMD6 deletions/amplifications and expression levels whereas analyses of the TCGA-GBM data through OASIS (http://www.oasis-genomics.org/) indicated that FRMD6 is deleted in 3.3% (19/577) of GBM cases and in 0.8% (3/371) cases of lower grade glioma. In addition, 8% (4/50) of glioma cell lines have FRMD6 deletion (Table 1), which is consistent with the potential tumor suppressor function of FRMD6 in GBM.

**Table 1:** Gene report_FRMD6

Project	Tissue	Somatic Mutation	Copy Number Gain	Copy Number Loss
A. Alteration Summary_Cancer Projects (Donors Affected/Donors Analyzed)				
Brain lower grade glioma (TCGA, US)	Glioma	0.4% (1/227)	(0/433)	0.8% (3/371)
Glioblastoma multiforme (TCGA, US)	Glioblastoma	0.5% (2/396)	(0/1076)	3.3% (19/577)

B. Alteration Summary_CCLE Cell Lines				
Cancer or Tissue Type	Substitution	Indel	Copy Number Gain	Copy Number Loss
Brain-glioma	0%	0%	0%	8% (4/50)

Data derived from http://www.oasis-genomics.org/ (http://www.oasis-genomics.org/martreport/?report=report&mart = gene_report&ensembl_gene_id = ENSG00000139926&datasets=hsapiens_gene_ensembl_oasis1hkugc).

**Figure 1:**
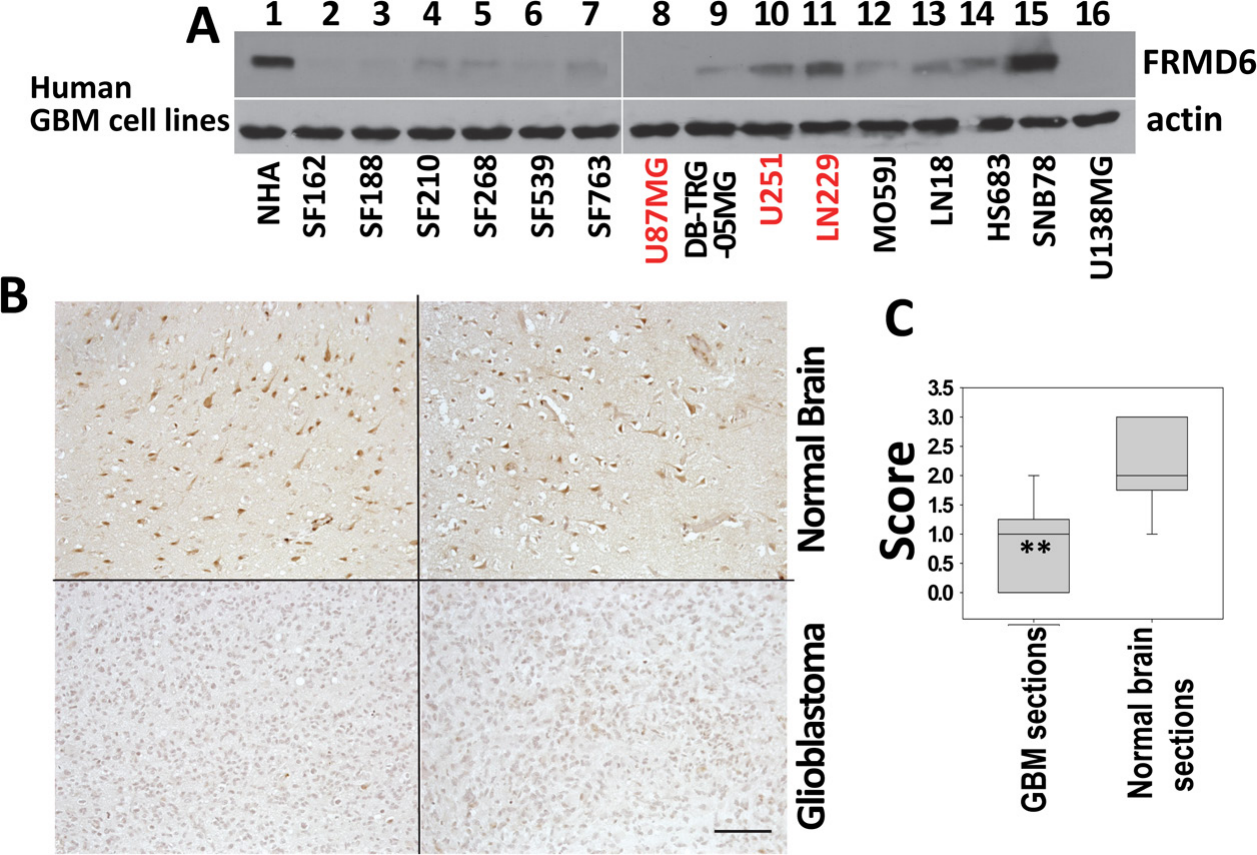
FRMD6 is down-regulated in human glioblastoma cells and tissues (**A**) Levels of endogenous FRMD6 protein in a panel of human GBM cells and normal human astrocytes (NHAs, ScienCell Research Laboratories) were determined by Western blotting using anti-FRMD6 antibody (Sigma, upper panels). 50 μg of proteins were loaded into each lane. Actin was included as an internal control for loading (bottom panels). (**B**) Immunohistochemistry (IHC) analyses were performed on 10 human GBM samples and 10 normal human brain samples using anti-FRMD6 antibody (Sigma). Representative pictures of the antibody staining patterns of FRMD6 in normal brain (upper two panels) and GBM tissues (bottom two panels) are shown. Bar, 150 μm. (**C**) Scoring of the IHC results: Intensity of immunoreactivity to anti-FRMD6 antibody in 10 GBM and 10 normal brain cases were scored as the following: score 0 = negative, 1 = weak, 2 = intermediate, and 3 = strong staining. The scores were averaged and standard deviations and *p* values were calculated. **p < 0.01.

To further determine FRMD6 protein levels in GBM cells and tissues, we performed western blotting and immunohistochemistry analyses, respectively, of protein samples derived from human GBM cells and normal human astrocytes (NHAs) and paraffin sections of human GBM tumors and normal human brain tissues. Our results showed that in general GBM cells and tissues express lower levels of FRMD6 comparing to normal human astrocytes and normal brain tissues (Figure 1).

### Increased expression of FRMD6 inhibits GBM cell proliferation and invasion

**Figure 2:**
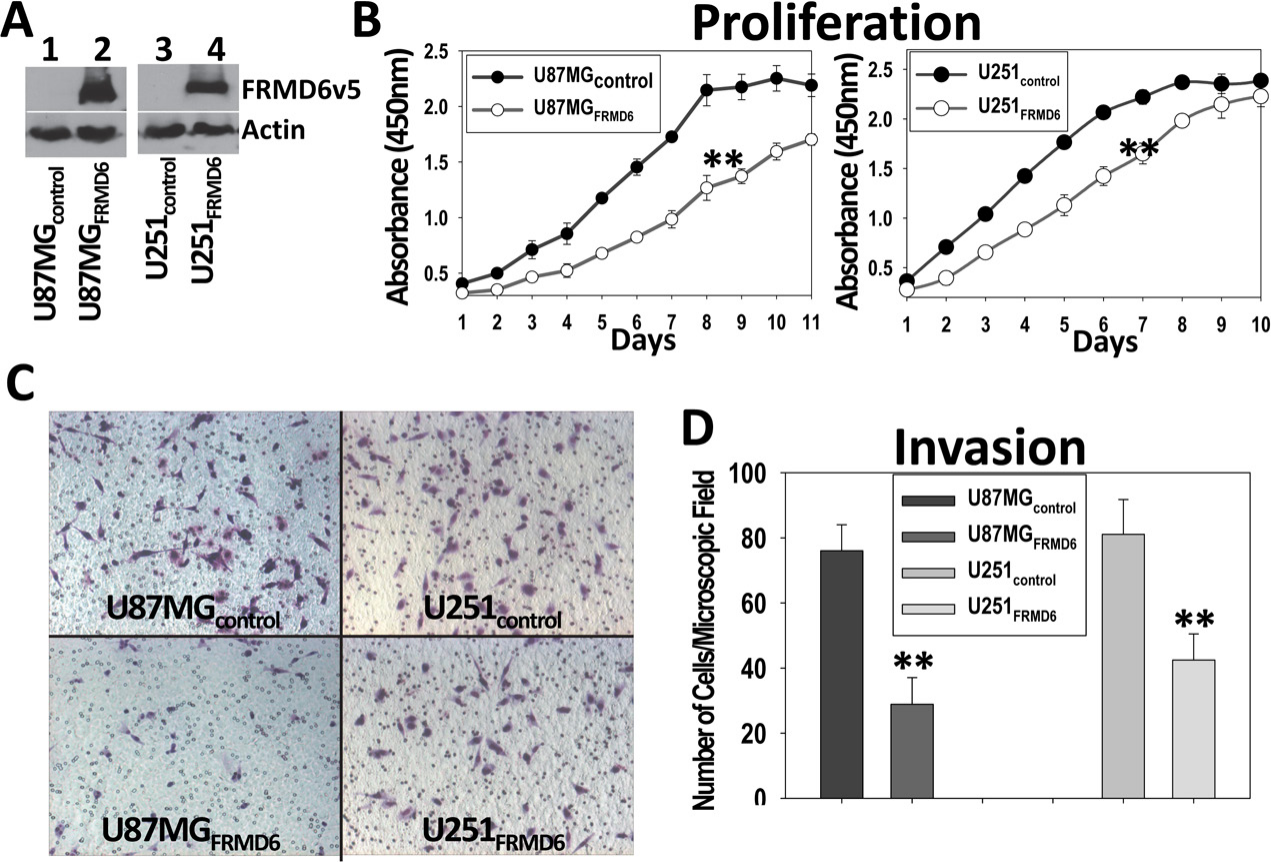
Increased expression of FRMD6 inhibits the GBM cell proliferation and invasion through Matrigel (**A**) Establishment of U87MG and U251 GBM cells expressing v5 epitope tagged FRMD6 (U87MG/U251_FRMD6_) or transduced with empty retroviral expression vector alone (U87MG/U251_control_). FRMD6_v5_ was detected by anti-v5 mAb (Invitrogen). (**B**) GBM cell proliferation assays were performed every day using a set of 96-well plates and the Premix WST1 kit (TaKaRa) following the manufacturer's instruction. **p < 0.01. (**C**) Invasion capacity of the transduced GBM cells was assessed by using the Matrigel (Corning) coated transwell plates. Representative images of the GBM cells migrated through the Matrigel-coated transwell inserts are shown and the round holes represent 8-μm pores on transwell membranes. (**D**) GBM cells migrated through the Matrigel-coated transwell inserts in 30 random selected 100× microscopic filed were counted and their quantitative mean values with SD are shown. **p < 0.01.

Among the GBM cells listed in Figure 1A, U87MG and U251 are highly tumorigenic and LN229 and SF763 are tumorigenic in immunocompromised mice. To assess the effect of FRMD6 on the GBM cells, we transduced U87MG and U251 cells that express little or a detectable level of endogenous FRMD6 (Figure 1A), respectively, with retroviruses carrying empty retroviral expression vector (U87MG/U251-control) or FRMD6 expression construct (U87MG/U251-FRMD6). After selection of the infected cells with puromycin, pooled populations of the drug-resistant U87MG/U251 cells express v5-epitope tagged FRMD6 (FRMD6v5, Figure 2A) at the levels that are similar to those of endogenous FRMD6 expressed by normal human astrocytes or certain GBM cells (Figure 1A, lane 1, 15). Cytoplasmic and nuclear location of FRMD6 was revealed by immunocytochemistry analysis of GBM cells expressing v5-tagged FRMD6 protein (Supplementary Figure S1). To determine how FRMD6 affects the GBM cellular behaviors, we investigated its effect on GBM cell proliferation and invasion. We found that increased expression of FRMD6 significantly inhibits GBM cell proliferation and invasion through Matrigel (Figure 2B–2D), suggesting that FRMD6 displays anti-GBM activity *in vitro*.

### FRMD6 inhibits subcutaneous and intracranial growth of GBM cells

**Figure 3:**
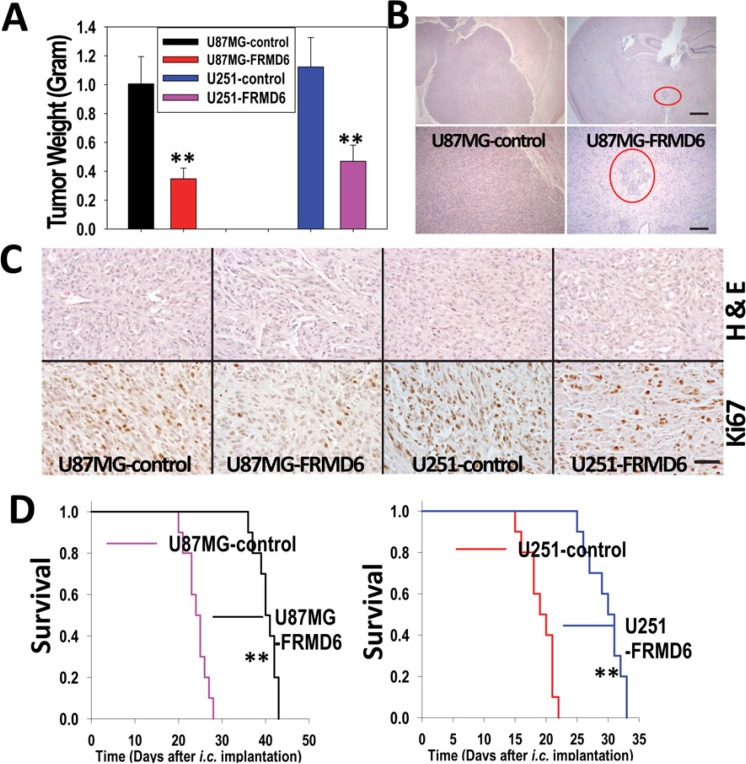
Increased FRMD6 expression inhibits subcutaneous and intracranial GBM growth and progression (**A**) 1 × 10^6^ U251 and 5 × 10^6^ U87MG GBM cells were injected subcutaneously into each immunocompromised Rag-2/II2rg mouse. Subcutaneous GBM growth experiments were terminated when the fastest growing gliomas reach ~1 cm in their longest diameters in accordance with the IACUC regulation and dissected subcutaneous tumors were weighted, recorded, and expressed as the mean weight +/− SD. *n* = 10 mice/group. **p < 0.01. (**B**) Representative images of the H&E stained cross sections of mouse brain bearing glioblastomas derived from U87MG-control (left two panels) and U87MG-FRMD6 (right two panels) cells 25 days after *i.c.* injection of the GBM cells. Bar is 40 μm in upper two panels and 160 μm in bottom two panels. Red circles in two right panels highlight a smaller glioblastoma derived from U87MG-FRMD6 cells. (**C**) Morphology and *in situ* anti-Ki67 antibody reactivity that highlights proliferating GBM cells in tumors are shown. The GBM sections were stained with H&E to outline histological morphology of the tumors (upper 4 panels). *In vivo* proliferating GBM cells were detected using an anti-Ki67 antibody (Fisher Scientific, bottom 4 panels). These GBM sections were derived from U87MG-control (first upper and bottom panels), U87MG-FRMD6 (second upper and bottom panels), U251-control (third upper and bottom panels), and U251-FRMD6 (last upper and bottom panels). Bar, 100 μm. (**D**) For intracranial GBM growth experiments, U87MG (3 × 10^5^ cells in 10 μl HBSS/Rag2 mouse)/U251 cells (2 × 10^5^cells in 10 μl HBSS/Rag2 mouse) were injected intracranially. *n* = 10 mice/group. Following the intracranial injections, mice were monitored closely and durations of their survival were recorded. Increased expression of FRMD6 was found to inhibit intracranial growth/progression of the GBM cells and extends survival of the experimental mice. **p < 0.01.

To determine how FRMD6 affects GBM growth and progression *in vivo*, pooled populations of U87MG and U251 cells transduced with empty expression vector or expressing v5-tagged FRMD6 (Figure 2A) were used in the *in vivo* subcutaneous (*s.c.*) and intracranial (*i.c*.) tumor growth experiments. The subcutaneous GBM growth experiments were terminated when the fastest growing gliomas reach ~1 cm in their longest diameters in accordance with the IACUC regulation and the intracranial GBM progression was monitored through the survival of their host mice. Our results show that increased expression of FRMD6 significantly inhibits the subcutaneous growth (Figure 3A, 3C) and intracranial progression (Figure 3B, 3D, Supplementary Figure S2) of GBMs and extends survival of experimental mice that were intracranially implanted with the GBM cells. To determine the cellular mechanism that underlies the anti-GBM effect of FRMD6, we analyzed proliferation status of the transduced U87MG/U251 cells *in vivo* and observed that increased expression of FRMD6 inhibits the GBM cell proliferation as these GBM cells display the reduced Ki67 reactivity *in situ* comparing to the control GBM cells (Figure 3C).

To further confirm the inhibitory effect of FRMD6 on GBM growth and progression, we assessed the FRMD6 effect on a newly established primary human GBM cell, WM47GBM. The STR DNA profile of WM47GBM cells was established by using the Power Plex16HS System by the LabCorp-Genetica at 15 STR loci and Amelogenin locus. After searching the STR databases of available cell lines in the ATCC, JCRB, and RIKEN repositories (http://www.atcc.org/str_database.aspx and https://www.dsmz.de/services/services-human-and-animal-cell-lines/online-str-analysis.html), we determined that WM47GBM is a unique new GBM cell (Supplementary Table S1). WM47GBM cells express little endogenous FRMD6 and are capable of forming intracranial tumors upon implantation (Supplementary Figure S3). Using pooled population of the transduced WM47GBM cells, we confirmed that increased expression of FRMD6 inhibits WM47GBM growth and progression *in vivo* and prolongs survival of the experimental mice implanted with intracranial WM47GBM cells (Supplementary Figure S3).

### Knockdown of FRMD6 expression promotes the GBM growth and progression *in vivo*

**Figure 4:**
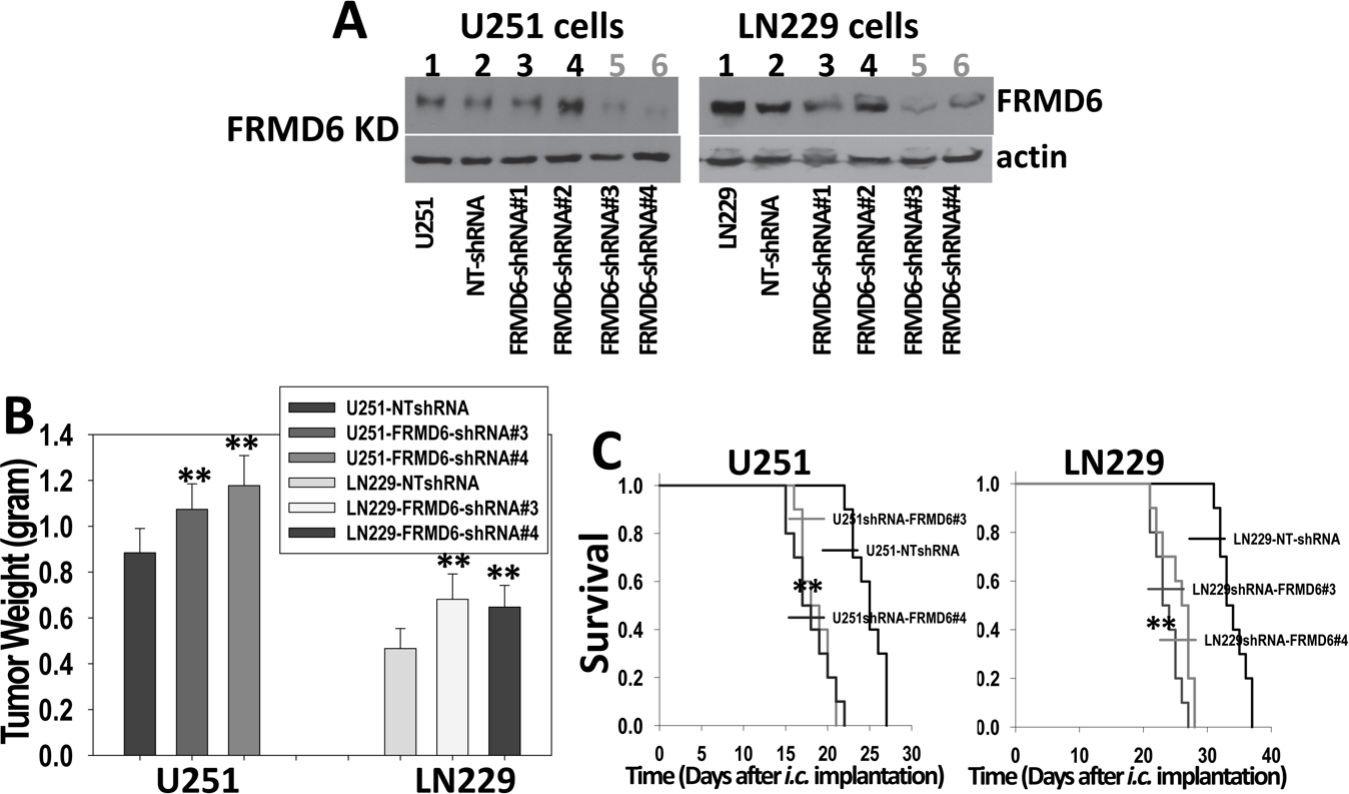
Knockdown of FRMD6 expression promotes growth and progression of GBM *in vivo* (**A**) Western blotting analyses show effective knockdown of FRMD6 in U251 (left panels) and LN229 (right panels) cells by shRNAs against human FRMD6 (Open Biosystems) whereas non-targeting (NT) control shRNA displays little effect. (**B**) 1 × 10^6^ transduced U251 and 5 × 10^6^ transduced LN229 GBM cells with (shRNA-FRMD6#3 and #4) or without (NTshRNA) FRMD6 knockdown were injected subcutaneously into each immuno compromised Rag-2/II2rg mouse. Subcutaneous GBM growth experiments were terminated when the fastest growing gliomas reach ~1 cm in their longest diameters and dissected subcutaneous tumors were weighted, recorded, and expressed as the mean tumor weight +/− SD. *n* = 10 mice/group. **p < 0.01. (**C**) Transduced U251 (1 × 10^5^cells in 10 μl HBSS/Rag2 mouse) and LN229 (4 × 10^5^cells in 10 μl HBSS/Rag2 mouse) cells with (shRNA-FRMD6#3 and #4) or without (NTshRNA) FRMD6 knockdown were injected intracranially. Mice were monitored closely and durations of their survival were recorded. Survival rates of mice following intracranial injections of the transduced U251 (left panel) and LN229 (right panel) cells with or without FRMD6 knockdown are shown. *n* = 10 mice/group. Knockdown of FRMD6 expression promotes intracranial growth of U251 and LN229 cells and extends survival of the experimental mice. **p < 0.01.

SNB78 GBM cells express a higher level of endogenous FRMD6 but display no tumorigenicity in immunocompromised mice (data not shown) and therefore, cannot be used to assess the effect of FRMD6 knockdown on GBM growth and progression. Unlike U87MG and WM47GBM cells that express little endogenous FRMD6, U251 and LN229 cells express detectable levels of endogenous FRMD6 (Figure 2A, lane 10 and 11). To determine whether knockdown (KD) expression of endogenous FRMD6 in U251 and LN229 cells affects GBM growth and progression, we first screened a set of shRNA constructs against human FRMD6 (Open Biosystems). A non-targeting (NT) shRNA construct was used as a control. Lenti-viruses carrying these shRNA constructs were used to infect U251 and LN229 cells. We found that two FRMD6 shRNA constructs (shRNA-FRMD6#3 and #4) reduces FRMD6 expression by at least 70% whereas the NTshRNA control construct displays little effect on FRMD6 expression in U251 and LN229 cells (Figure 4A).

We investigated the effect of FRMD6 knockdown on GBM cell proliferation and invasion and found that reduced FRMD6 expression significantly enhances GBM cell proliferation and invasion through Matrigel (Supplementary Figures S4 and S5), confirming that FRMD6 knockdown induces an effect opposite to that induced by increased FRMD6 expression. We then assessed tumorigenicity of pooled populations of U251/LN229-NTshRNA cells that serve as the controls, U251/LN229shRNA-FRMD6#3, and U251/LN229 shRNA-FRMD6#4 cells by injecting these cells subcutaneously or intracranially into immunocompromised Rag-2/II2rg mice. The subcutaneous GBM growth experiments were terminated when the fastest growing gliomas reach ~1 cm in their longest diameters in accordance with the IACUC regulation and dissected subcutaneous tumors were weighted and recorded. Since intracranial U251 GBMs progress quickly (Figure 3D, right panel), to better distinguish the potential pro-GBM growth effect of FRMD6 knockdown, we reduced the numbers of intracranially implanted U251 cells as detailed in figure legend (Figure 4). Our results showed that FRMD6 knockdown in U251 and LN229 cells significantly promotes subcutaneous GBM growth (Figure 4B) and intracranial GBM progression and reduces survival lengths of these mice carrying the intracranial tumors derived from U251/LN229shRNA-FRMD6#3or#4 cells comparing to the mice carrying intracranial tumors derived from U251/LN229-NTshRNA cells (Figure 4C, Supplementary Figure S6). These results are consistent with the notion that FRMD6 plays an important role in inhibiting GBM growth and progression and that loss of FRMD6 during the gliomagenesis and GBM progression is a critical step for the disease progression. Therefore, it is important to understand how FRMD6 exerts its anti-GBM activity and how reduced or lost expression of FRMD6 leads to the disease progression.

### Unlike increased merlin expression, increased FRMD6 expression has little effect on the stress-induced activation of the Hippo signaling pathway

**Figure 5:**
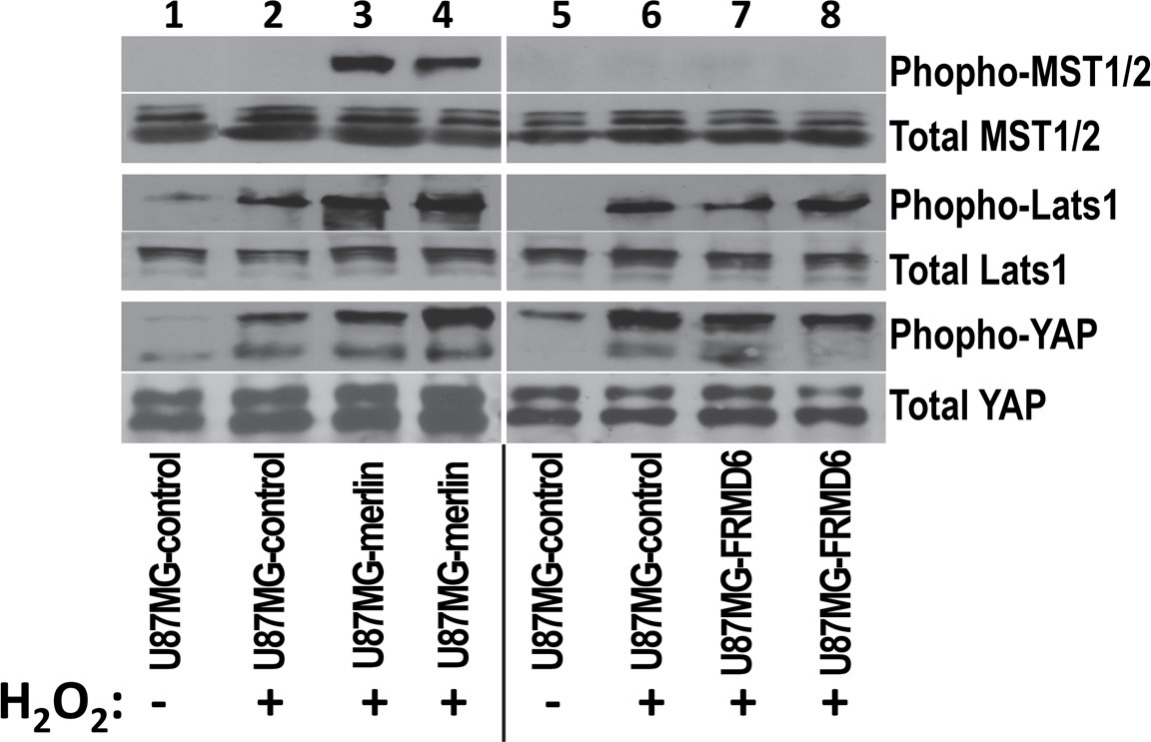
Unlike increased expression of merlin that enhances the H_2_O_2_-induced activation of the Hippo signaling pathway, increased expression of FRMD6 displays little effect Western blotting analyses were performed using the cell lysates derived from U87MG-control (lanes 1–2 and 5–6), U87MG-merlin (lanes 3–4), and U87MG-FRMD6 (lanes 7–8) cells. These cells were first treated with or without 60 μm H_2_O_2_ for 1hour as indicated in the panel. 50 μg of total proteins were loaded in each lane. Actin was included as an internal control for protein loading. Proteins were detected by different antibodies as outlined in right side of the panels.

*Drosophila* expanded (*ex*) has been shown to activate the Hippo signaling pathway [3, 11] and we established that increased expression of merlin enhances the stress-induced activation of the Hippo pathway in GBM cells [15]. To determine whether FRMD6 functions in a similar way to merlin in human GBM cells, we investigated the effect of FRMD6 on the H_2_O_2_-induced activation of the Hippo pathway. We found that unlike increased expression of merlin, which enhances the H_2_O_2_-induced activation of the Hippo pathway through increasing phosphorylation/activation of MST1/2 and Lats1 and phosphorylation/inactivation of YAP; increased expression of FRMD6 has little effect on the stress-induced activation of the Hippo components (Figure 5), suggesting that FRMD6 and merlin play a differential role in regulating the Hippo pathway in human glioblastoma.

### Increased expression of FRMD6 inhibits activities of several receptor tyrosine kinases (RTKs)

**Figure 6:**
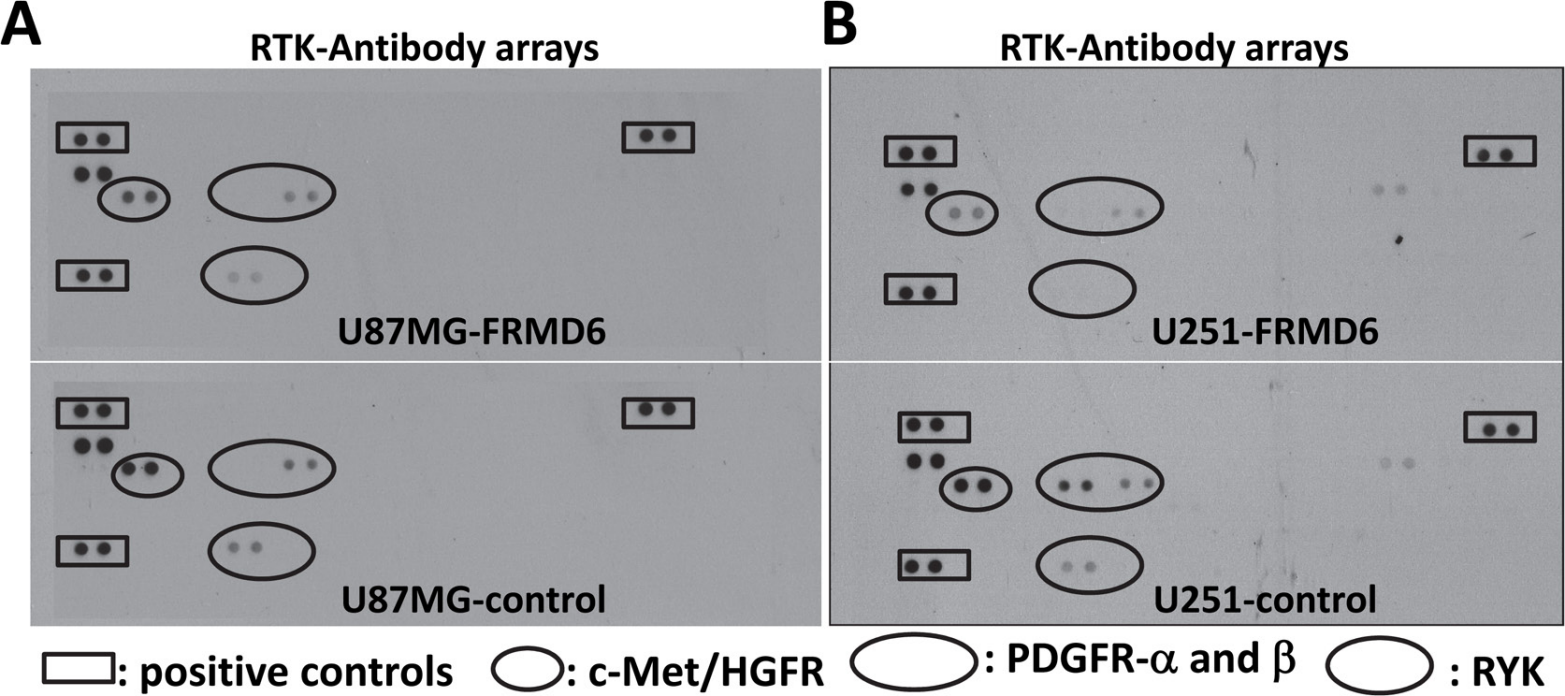
Increased expression of FRMD6 inhibits activities of several receptor tyrosine kinases (RTKs) (**A**–**B**) Equal amounts of protein lysates derived from U87MG-control (A-bottom panel), U87MG-FRMD6 (A-upper panel), U251-control (B-bottom panel) and U251-FRMD6 (B-upper panel) cells were applied to the Proteome Profiler Human Phospho-RTK Array membranes. Analyses of the phospho-receptor tyrosine kinase (RTK) arrays were performed following the manufacturer's instructions (R&D Systems). The squares and small/large/intermediate circles highlight positive controls, phospho-c-Met, phospho-PDGFRα/β, and phospho-RYK, respectively.

**Figure 7:**
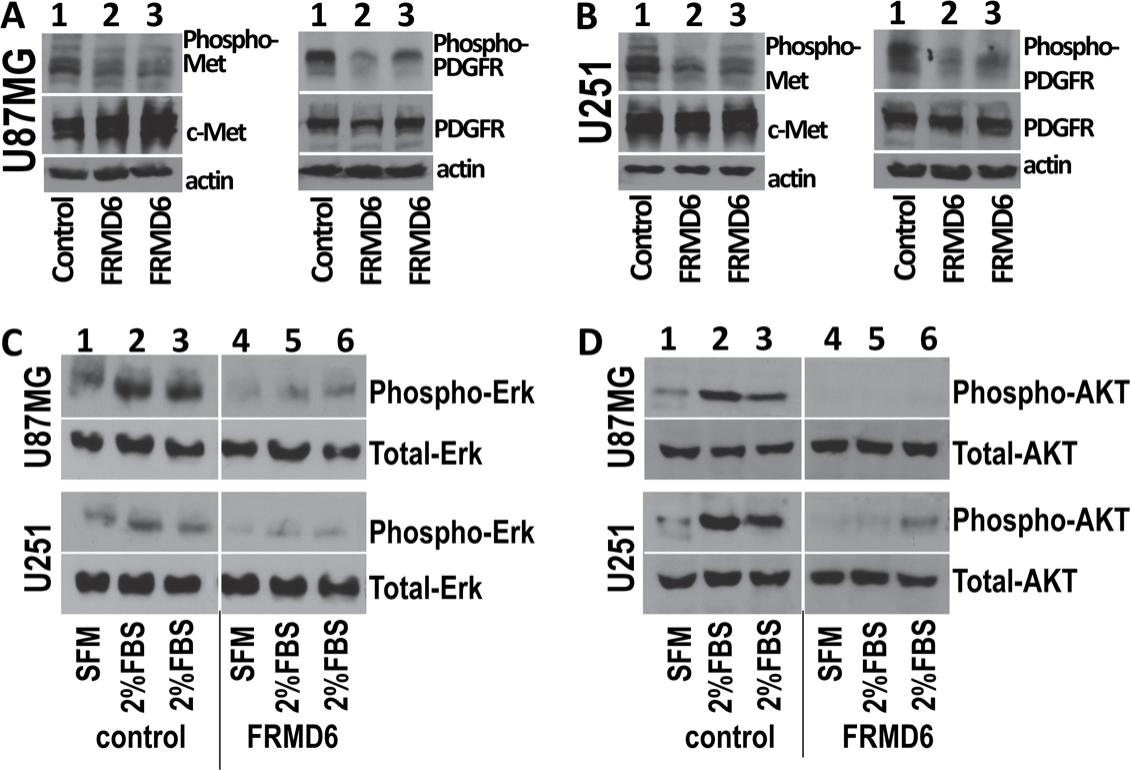
Increased expression of FRMD6 inhibits activities of c-Met and PDGFR RTKs and Erk and AKT kinases The Phospho-RTK Array results were confirmed by Western blotting analyses using the cell lysates derived from transduced U87MG cells (**A**, **C**, **D**) that express exogenous FRMD6 or transduced with empty expression vector (as control) and transduced U251 cells (**B**, **C**, **D**) that express exogenous FRMD6 or transduced with empty expression vector (as control) as labeled in the panels. 50 μg of proteins were loaded in each lane and actin was included as an internal control for protein loading. Anti-phospho-c-Met that detects activated c-Met, anti-c-Met that detects total c-Met, anti-PDGFRα+anti-PDGFRβ (PDGFRα/β), anti-phospho-PDGFRα/β, anti-phospho-Erk/AKT, and anti-Erk/AKT (Santa Cruz) were used as indicated in the panels.

To determine the mechanism underlying the anti-GBM effect of FRMD6, we performed antibody array analyses using the Proteome Profiler Human Phospho-RTK Array Kits (R&D Systems). Our results demonstrated that increased expression of FRMD6 reduces activity of c-Met, PDGFRα and/or β, and RYK RTKs in U87MG and U251 GBM cells (Figure 6A–6B, small, large and intermediate circles, respectively). To confirm these results, we assessed activity of c-Met and PDGFR in the GBM cells expressing high or low levels of FRMD6 by western blotting. The results confirmed that increased expression of FRMD6 reduces levels of phosphorylated/activated Met and PDGFRα/β in the GBM cells (Figure 7A–7B), suggesting that FRMD6 exerts its anti-GBM effect through or partially through inhibiting c-Met and/or PDGFR activity. RYK is an atypical member of the RTK family and is involved in the Wnt signaling [16]. Unfortunately, no commercial anti-phospho-RYK antibody is being sold separately and not in the RTK-array format to allow us to confirm the inhibitory effect of FRMD6 on RYK activity.

We then assessed the potential correlation between FRMD6 levels and activity of c-Met and PDGFR in GBM cell lines. Our results showed that in general the cells expressing high levels of FRMD6 display lower levels of phospho-c-Met and phospho-PDGFR (Supplementary Figure S7). On the other hand, GBM cells that have lower levels of FRMD6 do not always have higher levels of phospho-c-Met and phospho-PDGFR as these cells express different levels of endogenous c-Met and PDGFR and likely have other molecular events that could inhibit and/or activate c-Met/PDGFR activity in these GBM cells.

MAPK/Erk and PI3K/Akt are well established signaling components at downstream of RTKs [17]. To further investigate the inhibitory effect of FRMD6 on the c-Met/PDGFR signaling pathway, we assessed the effect of increased FRMD6 expression on the fetal bovine serum (FBS)-induced activation of Erk1/2 and AKT. Our results show that increased FRMD6 expression reduces the baseline activity of Erk1/2 and AKT and diminishes the FBS-induced activation of Erk1/2 and AKT kinases in GBM cells (Figure 7C–7D), further supporting the inhibitory effect of FRMD6 on activity of c-Met/PDGFR RTKs. In addition, we examined activity of Erk and ATK kinases and c-Met RTK on the GBM tumor sections derived from the GBM tumors with high or low FRMD6 levels by immunohistochemistry (IHC). Our results showed that increased FRMD6 expression reduces levels of phosphorylated/activated Erk, AKT, and c-Met (Supplementary Figure S8), which is consistent with the notion that FRMD6 inhibits activity of c-Met RTK and its downstream Erk and AKT kinases.

### FRMD6 exerts its anti-GBM effect largely through negatively regulating c-Met RTK activity

**Figure 8:**
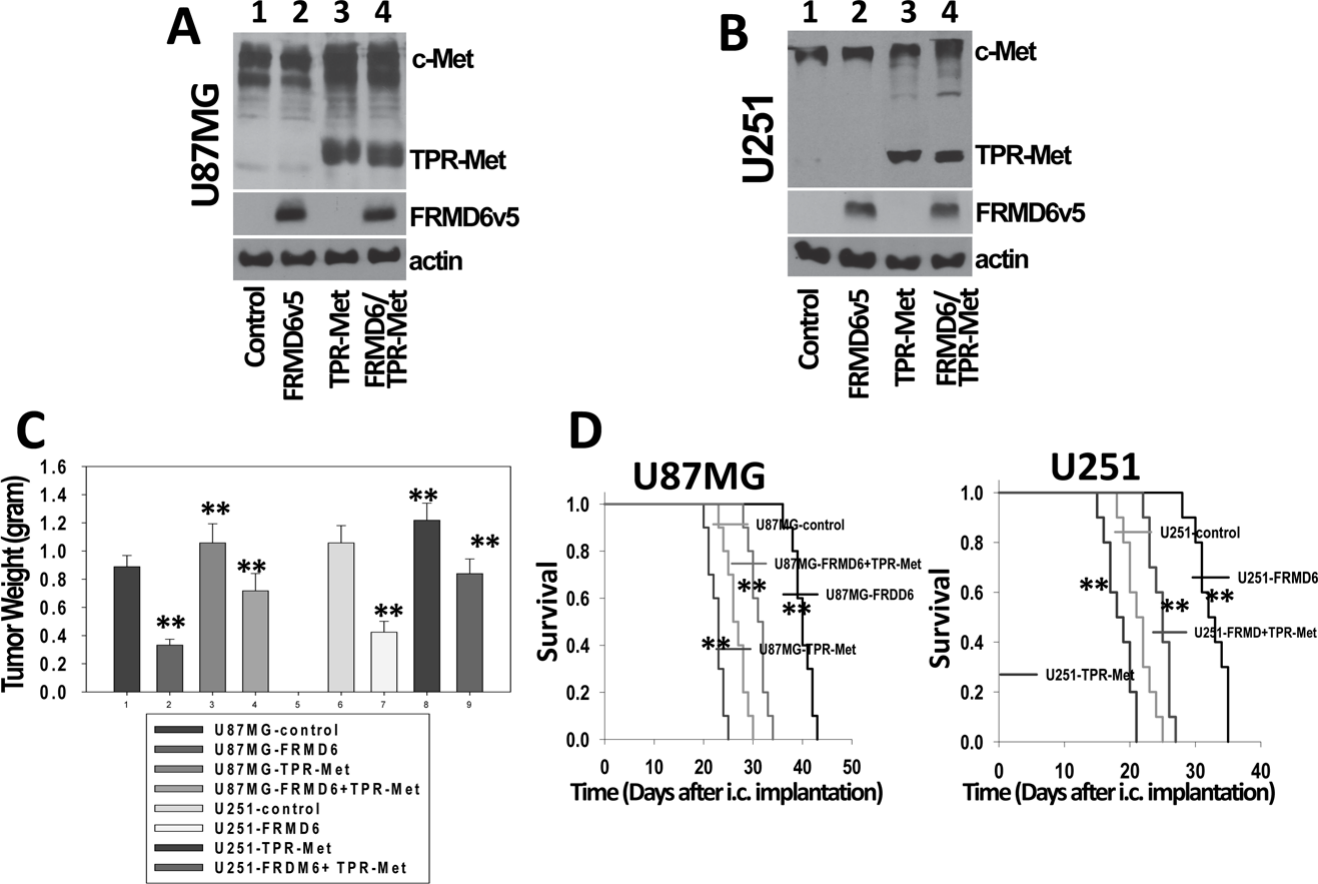
FRMD6 exerts its anti-GBM effect largely through negatively regulating c-Met RTK activity (**A**–**B**) Establishment of transduced U87MG (A) and U251 (B) cells that express TPR-Met fusion protein and/or v5-tagged FRMD6. In lane 1–4, protein lysates were derived from U87MG/U251 cells transduced with retroviruses carrying two different empty retroviral vectors or retroviruses carrying the expression constructs of FRMD6v5, TPR-Met, or TPR-Met plus FRMDv5, respectively. v5-tagged FRMD6 is in the vector containing the hygromycin-resistant gene whereas TPR-Met is in the vector containing the puromycin-resistant gene. 50 μg of proteins were loaded in each lane in A-B and actin was included as an internal control for protein loading. (**C**) Transduced 1 × 10^6^ U251 and 5 × 10^6^ U87MG GBM cells were injected subcutaneously into each immunocompromised Rag-2/II2rg mouse. Subcutaneous GBM growth experiments were terminated when the fastest growing gliomas reach ~1 cm in their longest diameters and dissected subcutaneous tumors were weighted, recorded, and expressed as mean weight +/− SD. *n* = 10 mice/group. **p < 0.01. (**D**) Transduced U87MG (3 × 10^5^ cells in 10 μl HBSS/Rag2 mouse)/U251 cells (2 × 10^5^cells in 10 μl HBSS/Rag2 mouse) were injected intracranially. Following the intracranial injections, mice were monitored closely and durations of their survival were recorded. Survival rates of experimental mice injected intracranially U87MG (left panel) and U251 (right panel) cells transduced with empty expression vectors or expressing FRMD6 and/or TPR-Met are shown. Ten mice were used for each type of transduced GBM cells in panel C–D. **p < 0.01.

c-Met is one of the predominant RTKs expressed by many GBM cells including U87MG and U251 cells (Supplementary Figure S7). In addition, c-Met activity can be easily manipulated. To determine whether FRMD6 exerts its anti-GBM effect through or partially through inhibiting c-Met RTK activity, we investigated whether expression a constitutively active c-Met kinase, the TPR-Met fusion protein [18], reverses or partially reverses the anti-GBM effect of FRMD6. The approximate 65 kDa cytoplasmic TPR-Met fusion protein is known to form a dimer through the leucine zipper of TPR and display constitutively activated c-Met kinase activity [18–20]. Retroviruses carrying the pBABE-Puro-TPR-Met expression construct (Addgene) or empty expression construct were transduced into the infected U87MG/U251 cells that express v5-tagged FRMD6 carried by retroviral expression construct containing hygromycin-resistant gene. We confirmed that the double hygromycin and puromycin-resistant U87MG and U251 cells express ~65 kDa TPR-Met and v5-tagged FRMD6 (Figure 8A–8B). These transduced GBM cells along with the controls were used in the subcutaneous and intracranial GBM growth/progression experiments. Our results show that expression of TPR-Met fusion protein largely overcomes the FRMD6-mediated anti-GBM effect *in vivo* (Figure 8C–8D), indicating that FRMD6 exerts its anti-GBM effect at least partially through inhibiting c-Met RTK activity. This finding established a novel mechanism underlying the novel inhibitory FRMD6 effect on GBM growth and progression.

## DISCUSSION

*Expanded* functions in parallel of *Drosophila* merlin (*mer*) and activates the Hippo signaling pathway [3]. Two previous studies showed that FRMD6 functions either through or independently of the Hippo pathway [1, 11]. We established that the anti-GBM effect of FRMD6 is not mediated through activating the Hippo signaling pathway but exerted through inhibiting activity of RTKs especially that of c-Met as the TPR-Met fusion protein largely reverses the anti-GBM activity of FRMD6 (Figures 5–8).

Our conclusion is supported by the sequence and structure differences between *Drosophila Expanded* and human FRMD6. *FRMD6* lacks the COOH-terminal sequences that are present in *Drosophila Expanded*. The corresponding COOH-terminal domain of Expanded contains the “PPXY” motifs, which is known to mediate the formation a complex with Yorkie, a *Drosophila* homolog of YAP. *EX* is thought to directly regulate Yorkie activity by interacting with the WW domains of Yorkie through its PPXY motifs [21]. It is conceivable that FRMD6 is unable to interact with YAP and functions independent of the Hippo pathway.

Both c-Met and PDGFR are often hyperactivated in GBM and known to drive the disease progression [17, 22, 23]. c-Met and PDGFR signaling pathways are known to promote GBM cell proliferation and motility [24, 25] and therefore, it is conceivable that reduction of c-Met and PDGFR activity by FRMD6 leads to the reduced GBM cell proliferation/invasiveness and GBM growth/progression. As a member of the band 4.1 family, FRMD6 contains the NH2-terminal “FERM (Four-point-one, ezrin, radixin, moesin)” domain, which is known to mediate the interactions between the band 4.1 family proteins and the cytoplasmic tails of transmembrane proteins [26, 27]. For example, merlin is known to negatively regulate EGFR activity [28] and CD44 function [29] through its FERM domain.

We attempted to determine whether FRMD6 and c-Met interact with each other by immunoprecipitation (IP). Unfortunately, FRMD6 is insoluble in the RIPA (radioimmunoprecipitation assay) buffer that contains 1%TritonX-100 and is frequently used in IP experiments. FRMD6 is soluble in 2% or 4% SDS sample buffer (Supplementary Figure S9), which makes it difficult to perform IP experiments. To provide some understanding of the potential mechanism by which FRMD6 might inhibit c-Met activity, we investigated their potential co-localization in GBM cells and found that FRMD6 is co-localized with c-Met in nuclei of GBM cells (Supplementary Figure S10, as showing yellow color and pointed by short white arrows). Studies have demonstrated the nuclear localization of the cytoplasmic fragments of c-Met in aggressive cancer cells [30] and that nuclear localized c-Met can initiate calcium signals [31]. RTKs or their cytoplasmic fragments have been found to travel to the nuclei through different mechanisms and the events are thought to regulate gene expression and signaling pathways [32, 33]. Co-localization of FRMD6 and c-Met in the GBM nuclei suggests that FRMD6 may negatively regulate c-Met functions in the GBM nuclei.

## MATERIALS AND METHODS

### Patient glioblastoma samples and reagents

Human GBM and normal brain tissues were obtained from the Cooperative Human Tissue Network (CHTN). Anti-FRMD6 (Sigma), -Lats1/2 (Bethyl Lab), -MST1/2, -YAP, -c-Met, -PDGFRA, -PDGFRB, and -merlin (Santa Cruz), -actin (Sigma), -v5 epitope (Invitrogen), -phospho-Lats1, -phospho-YAP (Cell signaling), -phospho-c-Met (Invitrogen and Santa Cruz), and -phospho-PDGFRA/B (Santa Cruz and R & D Systems) antibodies were used in the experiments. Anti-Ki67 was from the Fisher Scientific. Secondary anti-rabbit Alexa Fluor^®^ 594 and anti-mouse Alexa Fluor^®^ 488 were from Invitrogen.

### Cells, GBM cell lines, and primary GBM cells

Normal human astrocytes (NHAs) were obtained from ALLCELLS, Inc and ScienCell. SF126, SF188, SF210, SF295, and SF763 cells were from the UCSF Neurosurgery Tissue Bank and these cells were authenticated by the tissue bank at 15 Short Tanden Repeat (STR) loci plus Amelogenin locus using the PowerPlex16 System (Promega Corp.). U251 cells were from the DTP/DCTD NCI Tumor Repository and authenticated using the Applied Biosystems AmpFISTR Identifiler. U87MG and LN229 cells were obtained from the American Type Culture Collection (ATCC) and authenticated at 17 STR loci plus Amelogenin locus using Promega's PowerPlex^®^ 18D System. These cells were cultured and frozen down according to the providers' and manufacturers' instructions.

WM47 primary GBM cells were established from a fresh human GBM tissue provided by the CHTN and cultured in RPMI medium containing 10% FBS. The STR DNA profile of early passage WM47GBM cells were obtained by using the Power-Plex16HS System by the LabCorp-Genetica at 15 STR loci (Penta E, D18S51, D21S11, TH01, D3S1358, Penta D, CSF1PO, D16S539, D7S820, D13S317, D5S818, FGA, TPOX, D8S1179, and vWA) and Amelogenin locus. Based on analyses of the STR profiles of 8 STR and amelogenin loci that are available in the cell line databases of the ATCC, JCRB, and RIKEN repositories (http://www.atcc.org/str_database.aspx and https://www.dsmz.de/services/services-human-and-animal-cell-lines/online-str-analysis.html), the STR profile of WM47GBM cells does not match any known cell lines in these repositories, indicating that WM47GBM cell is a unique new human GBM cell (Supplementary Table S1). All the experiments were carried out using above mentioned GBM cells that were cultured less than 6 months after assessing their STR profiles or obtaining from different sources.

### Reverse transcriptase-polymerase chain reaction (RT-PCR), expression and knockdown constructions, and retrovirus transduction

RT-PCR was performed and full-length human FRMD6 cDNA was generated and cloned into the TA cloning vector (Invitrogen), which in turn re-cloned along with their COOH-terminal v5-epitope tags into retroviral expression vectors, pQCXIP or pQXIH (BD Bioscience) as described [34, 35]. The expression constructs were verified by DNA sequencing. pBABE-puroTPR-Met retroviral expression construct that carries a constitutively active cytoplasmic form of c-Met was obtained from the Addgene [18]. Several shRNA constructs against human FRMD6 and a non-targeting (NT) shRNA control were obtained from the Open Biosystems and Addgene. Retroviruses were generated using these expression or shRNA constructs following the manufacturer's instructions (BD Bioscience).

### Western blot analysis, phospho-receptor tyrosine kinase (RTK) array, immunocyto chemistry, and immunohistochemistry

Western blots were performed as described [34–36]. Briefly, cells were extracted with 4 x SDS Laemmli sample buffer without dye and protein concentrations were determined using Bio-Rad Dc Protein Assay Reagents. Phospho-receptor tyrosine kinase (RTK) Arrays were performed following the manufacturer's instruction (R&D Systems) using the Proteome Profiler Human Phospho-RTK Array Kits (R&D Systems).

Immunocytochemistry, histology, and immuno histochemistry were performed as described [34, 37]. Briefly, GBM cells were cultured in 35mm dishes at least 24 hours, and fixed with 3.7% paraformaldehyde. Fixed cells were permeabilized with 0.1% Triton X-100 made in PBS, washed with PBS, and blocked with 2% milk. The washed cells were then reacted with different antibodies and different fluorescence-conjugated secondary antibodies as detailed in figure legends. In addition, Paraffin sections derived from GBM patients and the GBM tumors plus adjacent normal mouse tissues derived from the *in vivo* subcutaneous and intracranial GBM growth experiments were stained with haematoxylin and eosin (H&E, Thermo Fisher) or reacted with different antibodies as detailed in figure legends. The intensity of immunoreactivity to anti-FRMD6 antibody was scored as the following: score 0 = negative, 1 = weak, 2 = intermediate, and 3 = strong staining following the established protocol [34, 37]. The scores were averaged and standard deviations and *p* values were calculated.

### Cell proliferation and invasion assays

Cell proliferation assays were performed by seeding the transduced GBM cells at 2 × 10^3^ cells/well in 96-well plates in triplicate in RPMI-1640 containing 10% FBS. Those cells were fed with fresh 10% FBS RPMI-1640 every day and the cell proliferation assays were performed using the Premix WST1 kit (TaKaRa) following the manufacturer's instruction.

Tumor cell invasion assays were performed using transwell chambers with inserts containing 8-μm pores (Corning) that were coated with a layer of Matrigel (Collaborative Biomedical) as described [14, 38]. Briefly, RPMI-1640 containing 10% FBS was added to bottom chambers of transwells. Transduced GBM cells (2–4 × 10^5^/well) were seeded onto the top cambers in triplicate and incubated for 24–48 h. Bottom of the inserts were then fixed and stained at end of experiments and the stained cells that had migrated through Matrigel were counted in 30 randomly selected 100× microscopic fields.

### Subcutaneous and intracranial tumor growth experiments

Mice were used in accordance with the approved IACUC Protocol. Pooled populations of the transduced U87MG, U251, LN229, and WM47GBM cells were used in subcutaneous and intracranial tumor growth experiments as described [37]. Briefly, 1–5 × 10^6^ GBM cells (for details, please see figure legends) were injected subcutaneously into each immuno-compromised Rag-2/II2rg mouse (Rag2; male or female, ~ 8 weeks of ago, Taconic, Hudson, NY). Each sets of the subcutaneous GBM growth experiments were terminated when the fastest growing gliomas reach ~1 cm in their longest diameters in accordance with the IACUC protocol and regulation and the dissected subcutaneous tumors were weighted and recorded. For intracranial tumor growth experiments, U87MG/WM47GBM/LN229 (2–4 × 10^5^cells in 10 μl HBSS/Rag2 mouse)/U251 cells (1–2 × 10^5^cells in 10 μl HBSS/Rag2 mouse) were injected as described [14, 37]. Numbers of GBM cells implanted intracranially were detailed in figure legends. Following the intracranial injections, mice were monitored closely and durations of their survival were recorded. Mice that showed signs of distress and morbidity were euthanized and considered as if they had died on that day. Survival rates were calculated as follows: survival = number of mice still alive/total number of experimental mice.

### Statistics

Other than survival experiments, student's *t* test was used to analyze statistical differences between the controls and experimental groups. For mouse survival experiments, the LogRank statistic analysis (SigmaPlot) was used. Differences were considered statistically significant at *p* < 0.05.

## SUPPLEMENTARY MATERIALS


