# Supplementary material for: FRMD6 inhibits human glioblastoma growth and progression by negatively regulating activity of receptor tyrosine kinases
Source: Oncotarget. 2016 Sep 20;7(43):70080–91. doi: 10.18632/oncotarget.12148 (PMC5342536; doi:10.18632/oncotarget.12148)
Supplement: Supplementary file 1 [file oncotarget-07-70080-s001.pdf]

FRMD6 inhibits human glioblastoma growth and progression by negatively regulating activity of receptor tyrosine kinases

Supplementary Materials

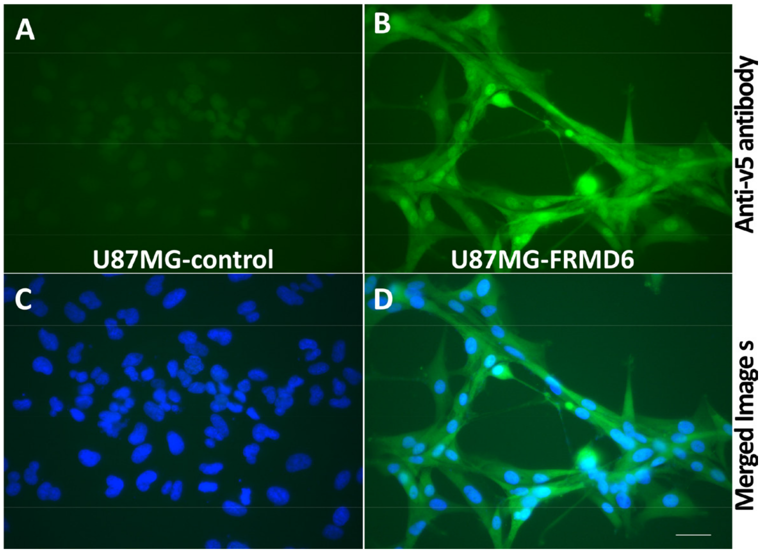

Supplementary Figure S1: Localization of FRMD6 in human glioblastoma cells. (**A–B**) Immunocytochemistry analyses were performed to localize v5-epitope tagged FRMD6 protein in U87MG-FRMD6 cells using anti-v5 mAb (Invitrogen) and FITC-conjugated secondary antibody (Sigma). (**C–D**) Merged images of the DAPI (diamidino-2-phenylindole)-highlighted nuclei (blue) and the reactivity of anti-v5-antibody plus FITC-conjugated secondary antibody (green) in U87MG-control and U87MG-FRMD6 cells are shown. Bar. 150 µm.

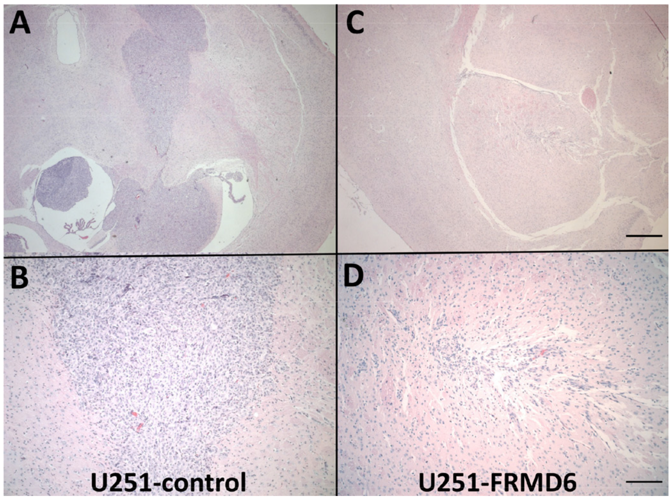

Supplementary Figure S2: Increased FRMD6 expression inhibits intracranial growth and progression of U251 cells. Representative images of the H&E stained cross sections of mouse brain bearing glioblastomas derived from U251-control (**A–B**) and U251-FRMD6 (**C–D**) cells 19 days after intracranial implantation of the GBM cells. Bar is 100 µm in upper two panels and 400 µm in bottom two panels.

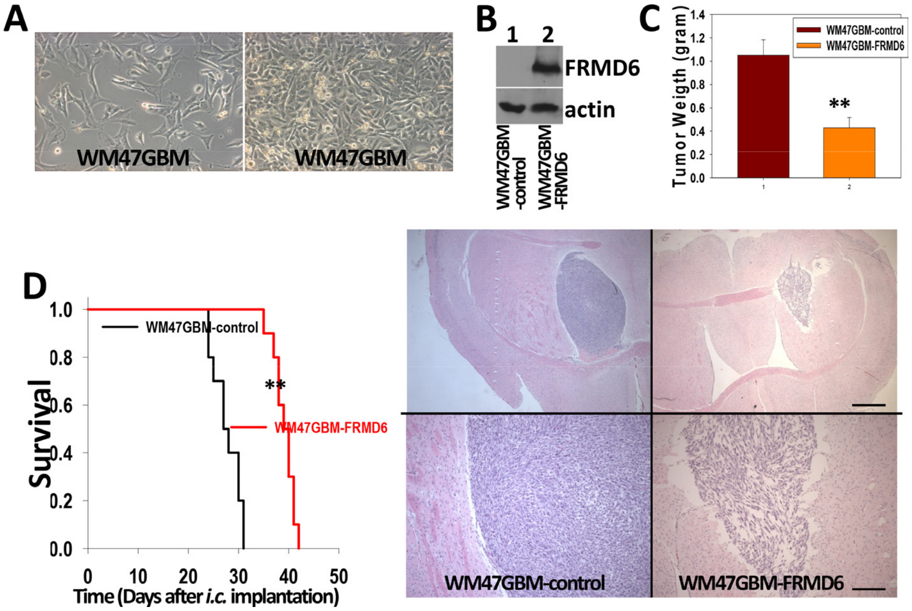

Supplementary Figure S3: Increased FRMD6 expression inhibits subcutaneous and intracranial growth and progression of WM47GBM cells. (**A**) Establishment of an unique new human primary GBM cell, WM47GBM. Phase pictures show WM47GBM cells grown in low (left panel) and high (right panel) density, respectively, in 10%FBS-RPMI medium. (**B**) Establishment of WM47GBM cells expressing v5 epitope tagged FRMD6 (WM47GBMFRMD6) or transduced with empty expression vector alone (WM47GBM-control). v5-tagged FRMD6 was detected by anti-v5 mAb (Invitrogen). (**C**) 2×10^6 WM47GBM cells were injected subcutaneously into each immunocompromised Rag-2/Il2rg mouse. Subcutaneous GBM growth experiments were terminated when the fastest growing gliomas reach ~1 cm in their longest diameters in accordance with the IACUC regulation and dissected subcutaneous tumors were weighted, recorded, and expressed as the mean weight +/− SD. n=10 mice/group. $**p < 0.01$. (**D**) WM47 GBM cells (2×10^5 cells in 10 µl HBSS/Rag2 mouse) were injected intracranially. $n = 10$ mice/group. Following the intracranial injections, mice were monitored closely and durations of their survival were recorded. Left Panel, increased expression of FRMD6 inhibits intracranial growth of the GBM cells and extends survival length of the experimental mice. $**p < 0.01$. Right Panel, representative images of the H&E stained cross sections of mouse brain bearing glioblastomas derived from WM47GBM-control (left two panels) and WM47GBM-FRMD6 (right two panels) cells 28 days after intracranial implantation of the GBM cells. Bar is 125 µm in upper two panels and 500 µm in bottom two panels.

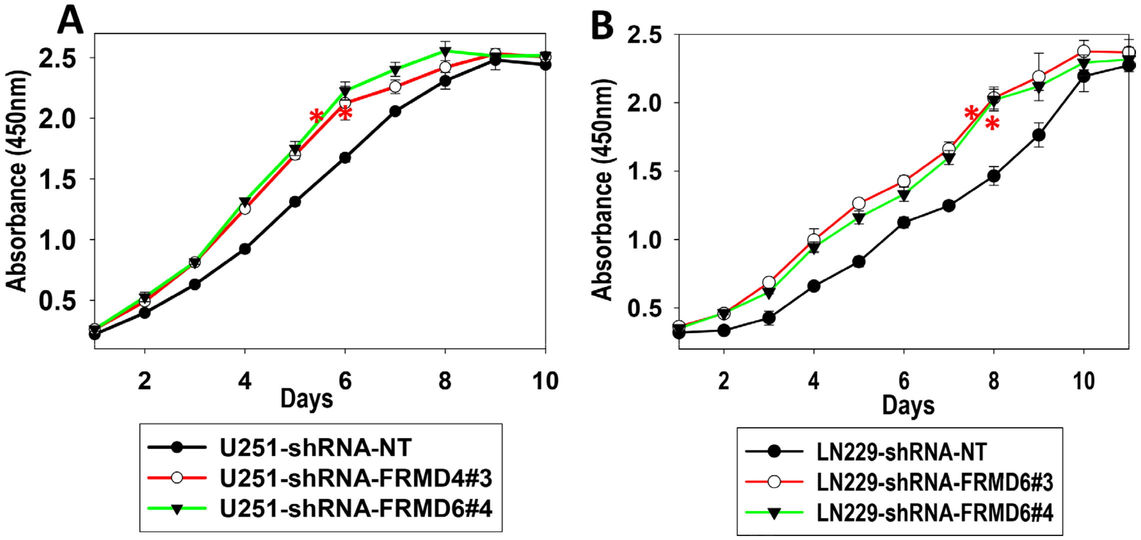

Supplementary Figure S4: Knockdown FRMD6 expression in U521 and LN229 cells promotes GBM cell proliferation. (**A–B**) Cell proliferation assays were performed by seeding transduced U251 (A) and LN229 (B) cells with or without FRMD6 knockdown at 2×10^3 cells/well in 96-well plates in triplicate. These cells were fed with fresh 10% FBS RPMI-1640 every day and the cell proliferation assays were performed every day using a set of 96-well plates and the Premix WST1 kit (TaKaRa) following the manufacturer's instruction. $**p < 0.01$.

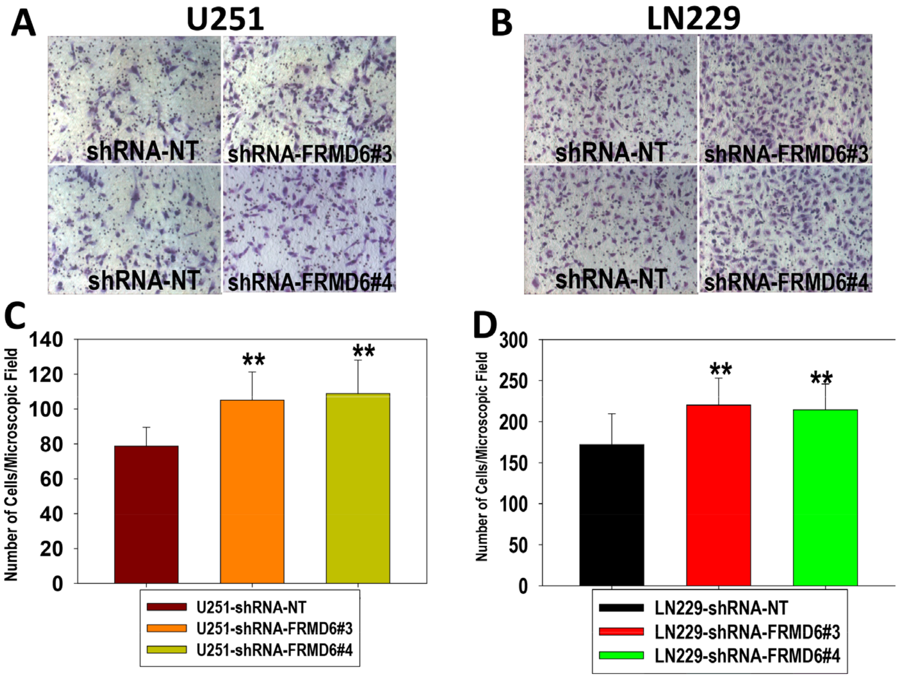

Supplementary Figure S5: Knockdown FRMD6 expression in U521 and LN229 cells promotes GBM cell invasion through Matrigel. Invasion capacity of the transduced U251 and LN229 cells with or without FRMD6 knockdown was assessed by using the Matrigel coated transwell plates. (**A**–**B**) Representative images of transduced U251 (A) and LN229 (B) cells migrated through the Matrigel-coated transwells are shown. (**C**–**D**) The transduced U251/LN229 cells migrated through the Matrigel-coated transwell inserts in 30 random selected 100× microscopic filed were counted and their quantitative mean values with SD are shown. $^{**}p < 0.01$.

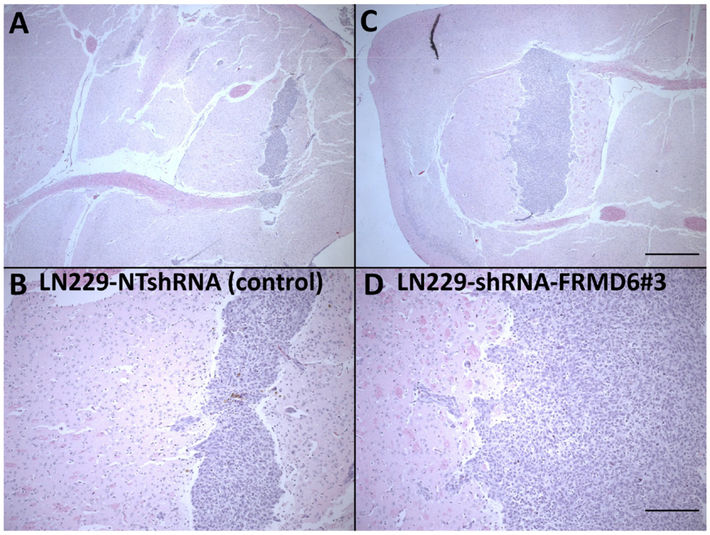

Supplementary Figure S6: Knockdown of FRMD6 promotes intracranial growth and progression of LN229 GBM cells. Representative images of the H&E stained cross sections of mouse brain bearing glioblastomas derived from LN229-NTshRNA (control) (**A**–**B**) and LN229-shRNA-FRMD6#3 (**C**–**D**) cells 24 days after intracranial implantation of the GBM cells are shown. Bar is 125 µm in upper two panels and 500 µm in bottom two panels.

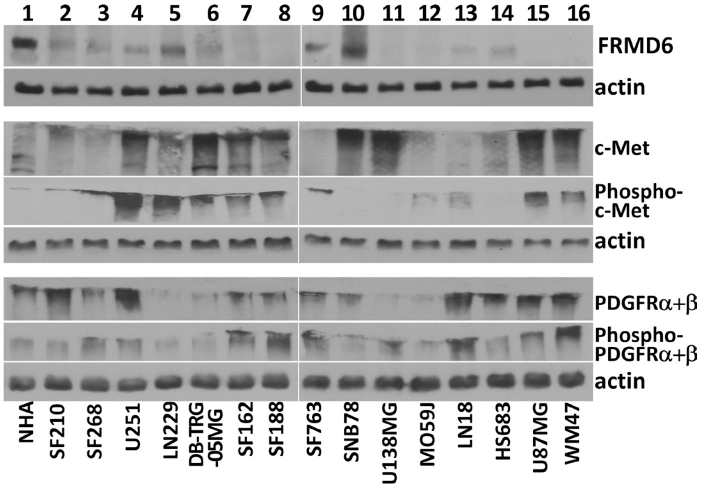

Supplementary Figure S7: Higher levels of FRMD6 are correlated with lower activities of c-Met/PDGFR in human glioblastoma cells. Levels of FRMD6, c-Met, phospho-c-Met, PDGFR, and phospho-PDGFR in a panel of human GBM cells and normal human astrocytes (NHAs) were determined by Western blotting using appropriate antibodies as labeled in the panels. 50 µg of proteins were loaded into each lane. Actin was included as an internal control for loading.

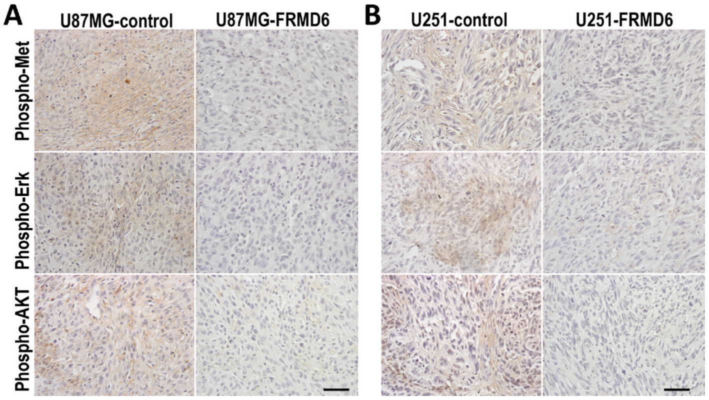

Supplementary Figure S8: Increased expression of FRMD6 inhibits activities of Erk1/2 and AKT kinases and c-Met RTK. GBM sections derived from U87MG-control (**A**, left panels), U87MG-FRMD6 (**A**, right panels), U251-control (**B**, left panels), and U251-FRMD6 (**B**, right panels) were reacted with anti-phospho-Erk, -AKT, or -c-Met antibodies as shown. Bar, 80 µm.

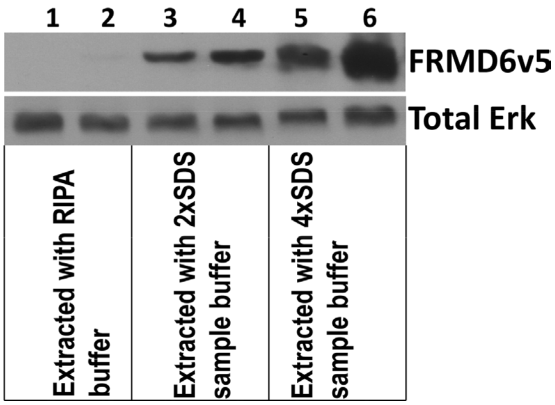

Supplementary Figure S9: FRMD6 protein is insoluble in the RIPA buffer. U251-FRMD6 (lanes 1, 3, 5) and U87MG-FRMD6 (lanes 2, 4, 6) cells were extracted with the RIPA buffer (lanes 1–2), 2 × SDS (lanes 3-4) or 4 × SDS (lanes 5–6) sample buffer. Western blotting analysis was performed using these GBM cell lystaes and anti-v5 antibody. 50 µg of proteins were loaded into each lane and total Erk1/2 was included as an internal control for loading.

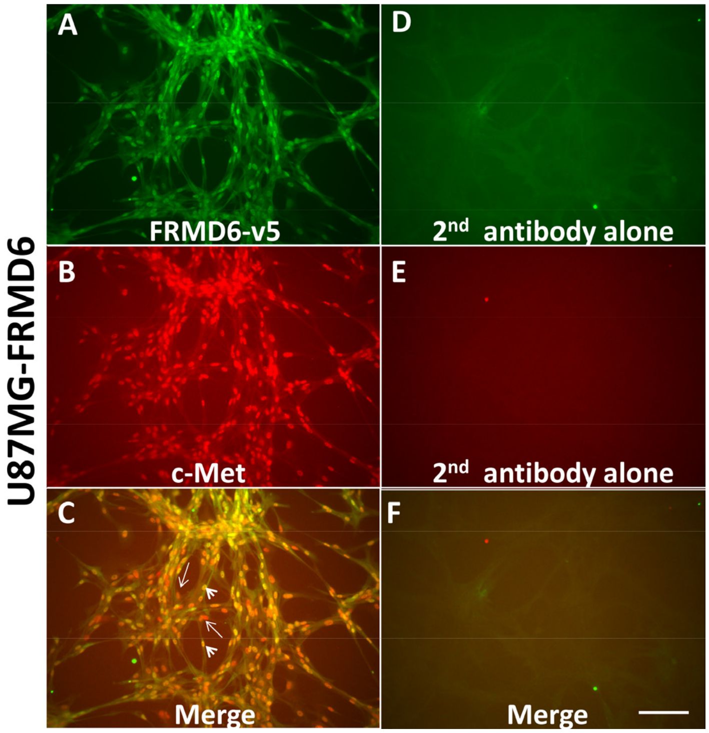

Supplementary Figure S10: Co-localization of FRMD6 and c-Met in the nuclei of human glioblastoma cells. Immunocytochemistry analyses were performed to localize v5-epitope tagged FRMD6 protein in U87MG-FRMD6 cells using anti-v5 mAb and anti-mouse Alexa Fluor® 488 (green color, **A**) and to localize c-Met protein using anti-c-Met antibody and anti-rabbit Alexa Fluor® 594 (red color, **B**). Merged images of the co-localized FRMD6v5 and c-Met in U87MG-FRMD6 cells that are highlighted in yellow color (**C**). U87MG-FRMD6 cells that only reacted with secondary antibodies are shown in (**D**, **E**, and **F**) as the controls. Bar. 40 µm.

Supplementary Table S1: The STR DNA profile establishes that WM47GBM cell is a unique and newly established primary human GBM cell

Gene/Loci	STR Allele Call(s)
AMEL	X, Y
D3S1358	16, 18
TH01	7, 9
D21S11	28
D18S51	12, 13
Penta_E	7, 13
D5S818	10, 12
D13S317	10
D7S820	9, 11
D16S539	8, 12
CSF1PO	10, 12
Penta_D	14, 15
vWA	14, 18
D8S1179	14
TPOX	8, 11
FGA	19, 24
